# Blockade of the Adenylate Cyclase Toxin Synergizes with Opsonizing Antibodies to Protect Mice against Bordetella pertussis

**DOI:** 10.1128/mbio.01527-22

**Published:** 2022-08-03

**Authors:** Andrea M. DiVenere, Dzifa Amengor, Rui P. Silva, Jory A. Goldsmith, Jason S. McLellan, Jennifer A. Maynard

**Affiliations:** a Department of Chemical Engineering, University of Texas, Austin, Texas, USA; b Department of Molecular Biosciences, University of Texas, Austin, Texas, USA; c LaMontagne Center for Infectious Disease, University of Texas, Austin, Texas, USA; Dartmouth College

**Keywords:** immune evasion, neutralizing antibodies, pediatric infectious disease, pertussis toxins

## Abstract

*Bordetella* produces an array of virulence factors, including the adenylate cyclase toxin (ACT), which is essential, immunogenic in humans, and highly conserved. Despite mediating immune-evasive functions as a leukotoxin, ACT’s potential role as a protective antigen is unclear. To better understand the contributions of humoral anti-ACT immunity, we evaluated protection against Bordetella pertussis by antibodies binding structurally defined ACT epitopes in a mouse pneumonia model. An ACT-neutralizing antibody, but not a nonneutralizing antibody or an isotype control, significantly increased mouse survival after lethal challenge with B. pertussis. When modified to impair Fc effector functions, the neutralizing antibody retained protective capabilities, indicating that protection was mediated by the blockade of the interactions of ACT with its α_M_β_2_ integrin receptor. After infection with a lower bacterial dose, ACT neutralization synergistically reduced lung bacterial colonization levels when combined with an opsonic antibody binding the surface antigen pertactin. Notably, protection was significantly enhanced when antibodies were administered intranasally as opposed to systemically, indicating that local immune responses are key to antibody-mediated protection against ACT and pertactin. These data reconcile previous conflicting reports to indicate that neutralizing anti-ACT antibodies support the phagocytosis of opsonized B. pertussis and thereby contribute to pertussis protection *in vivo*.

## INTRODUCTION

Diverse bacterial pathogens deploy cAMP-elevating toxins to help establish an infection. These include the delivery of enzymes that directly catalyze cAMP formation in the target cell and enzymes that elevate cAMP indirectly via altered G protein signaling (e.g., cholera toxin from Vibrio cholerae) and even the direct delivery of bacterium-generated cAMP (e.g., Rv0386 from Mycobacterium tuberculosis [1]). The edema factor of Bacillus anthracis and ExoY of Pseudomonas aeruginosa are two toxins that possess adenylate cyclase activities and are delivered into the target cell by different mechanisms. While neither of these is considered the major toxin produced by their respective bacteria, antibodies binding multiple edema factor epitopes protect against infection in animal models (2, 3), and two antibodies blocking P. aeruginosa type III secretion have been evaluated in human clinical trials (4, 5), supporting their roles as protective antigens.

The adenylate cyclase toxin (ACT) produced by Bordetella pertussis shares sequence and functional homologies with the adenylate cyclase domains of edema factor and ExoY despite delivery into target cells via a third mechanism. After binding leukocytes via the α_M_β_2_ integrin receptor (also called CR3, MAC-1, and CD11b/CD18), the N-terminal adenylate cyclase domain is directly translocated across the eukaryotic membrane. There, activated by calmodulin, ACT rapidly converts nearly all intracellular ATP to cAMP (6) to suppress local leukocyte antibacterial activities and thereby support bacterial colonization (7, 8). *Bordetella* strains with a natural or engineered reduction in ACT activity are less able to establish infection, especially during the early stages of colonization (9, 10), while some hypervirulent strains have elevated ACT expression (11). Moreover, ACT exhibits 98% sequence identity across strains and clinical isolates (12), and natural infection elicits strong anti-ACT antibody responses in humans (13, 14).

Despite being highly conserved, essential, and immunogenic, ACT’s role as a protective antigen and its potential to contribute to protection against pertussis have been disputed. Previous ACT immunization experiments provided conflicting evidence as to whether ACT contributes to protection in mice, with some reports observing only adjuvant effects that enhanced antipertactin responses (15, 16) and others observing reduced lung colonization levels attributable to anti-ACT antibodies (11, 17, 18). The complex organization of this large protein has contributed to the challenges in interpreting anti-ACT effects since antibodies binding different epitopes are likely to mediate different responses, and it is unclear which epitopes dominate after immunization. In addition to the N-terminal catalytic domain, ACT includes an acylated hydrophobic domain, which is likely involved in the translocation of the catalytic domain, and a C-terminal repeat-in-toxin (RTX) domain. Structurally, the RTX domain is comprised of five beta roll blocks joined by linkers that mediate receptor and antibody binding (19), with antibodies binding some linkers neutralizing ACT by sterically inhibiting RTX-receptor interactions (20, 21).

Here, we systematically evaluate the hypothesis that antibodies neutralizing ACT can ameliorate pertussis infection using a mouse pneumonia model of disease. Since ACT is thought to act in close proximity to a bacterium in the respiratory tract (22), we speculated that nasal delivery would result in higher antibody concentrations at the site of infection and would better mimic a strong mucosal immune response. Since ACT targets leukocytes to inhibit antibacterial activities, including phagocytosis and the respiratory oxidative burst (23), we further speculated that blockade of ACT function could support the clearance of opsonized bacteria. Accordingly, we evaluated the potential for ACT-neutralizing antibodies to synergize with opsonizing antibodies binding the current vaccine antigen pertactin. These experiments support the role of ACT as a protective antigen and suggest that vaccination strategies resulting in high levels of neutralizing anti-ACT responses in the respiratory mucosa will enhance protection by bactericidal antibodies against B. pertussis.

## RESULTS

### ACT supports respiratory tract colonization during B. pertussis infection.

**FIG 1:**
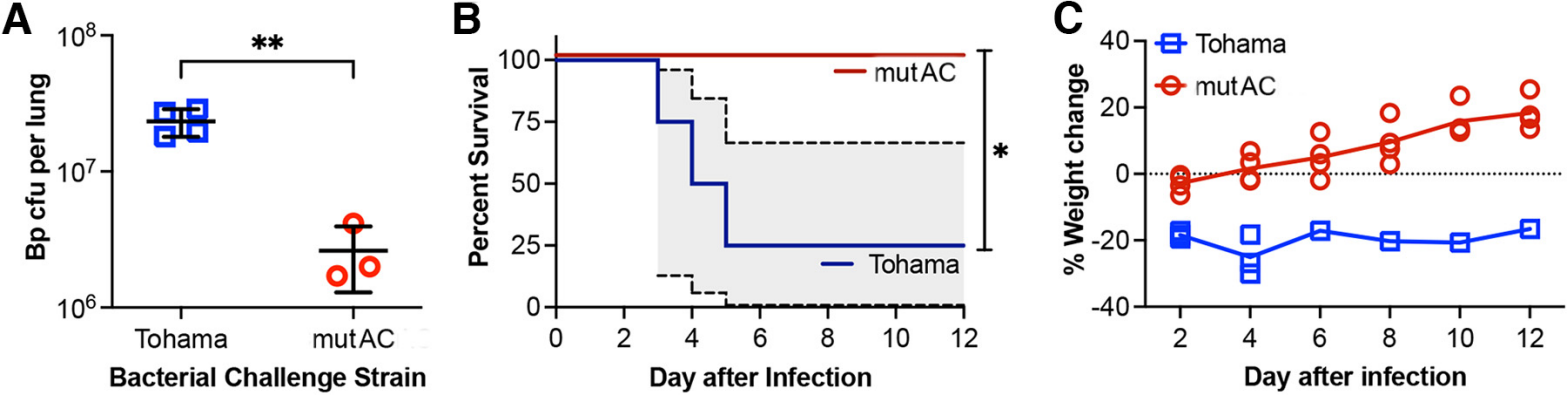
Impaired ACT activity reduces B. pertussis pathology in mice. (A) Bacterial lung colonization levels on day 3 after a low-dose infection (1 × 10^7^ CFU/mouse) with B. pertussis (Bp) expressing catalytically inactive ACT (TohamaI/mutAC [labeled mutAC]) versus TohamaI (labeled Tohama) (**, *P* < 0.01 [by a 1-tailed *t* test]) (*n* = 4 mice per group). (B) Mouse survival after a high-dose infection (3 × 10^8^ CFU per mouse) with B. pertussis TohamaI/mutAC versus the parental TohamaI strain (*, *P* < 0.05 [by Mantel-Cox test]) (*n* = 4 mice per group) (the 95% confidence interval is shown in gray). (C) Mouse weight changes after infection for mice shown in panel B. The experiment was repeated twice, with similar results.

To assess the impact of altered ACT activities on B. pertussis infection, we compared infections by B. pertussis TohamaI and an isogenic strain, TohamaI/mutAC, in which the ACT catalytic activity was disrupted by a dipeptide insertion between residues 188 and 189 (both generously provided by Peter Sebo) (10). Three days after a low-dose infection (1 × 10^7^ CFU/mouse), mice infected with TohamaI/mutAC had ~10-fold-fewer lung CFU than mice infected with TohamaI (*P* < 0.01) (Fig. 1A), consistent with the proposed role for ACT during the early stages of colonization. Day 3 was chosen since *cyaA* transcription levels increase ~1.5-fold by day 3 postinfection (24), and other reports have examined the impact of ACT on day 3, allowing comparisons among data sets (18).

A second experiment monitored survival after a high-dose infection with either strain (3 × 10^8^ CFU per mouse). Notably, 3 of 4 mice infected with TohamaI succumbed, compared to none of the mice infected with TohamaI/mutAC (*P* < 0.0001) (Fig. 1B). In both experiments, mouse weight provided a second metric of health, with rapid, extreme weight loss (>30%) correlating with morbidity. The lone surviving TohamaI-infected mouse experienced a sustained ~20% weight loss (Fig. 1C). These results are similar to those reported previously by Skopova et al. using identical strains (10) and Carbonetti et al. using a ΔACT strain (25). With a mouse model able to detect the impact of altered ACT activities on infection established, we proceeded to determine whether the passive administration of anti-ACT antibodies could capture these differences.

### Antibodies binding different RTX epitopes have similar biophysical characteristics.

**FIG 2:**
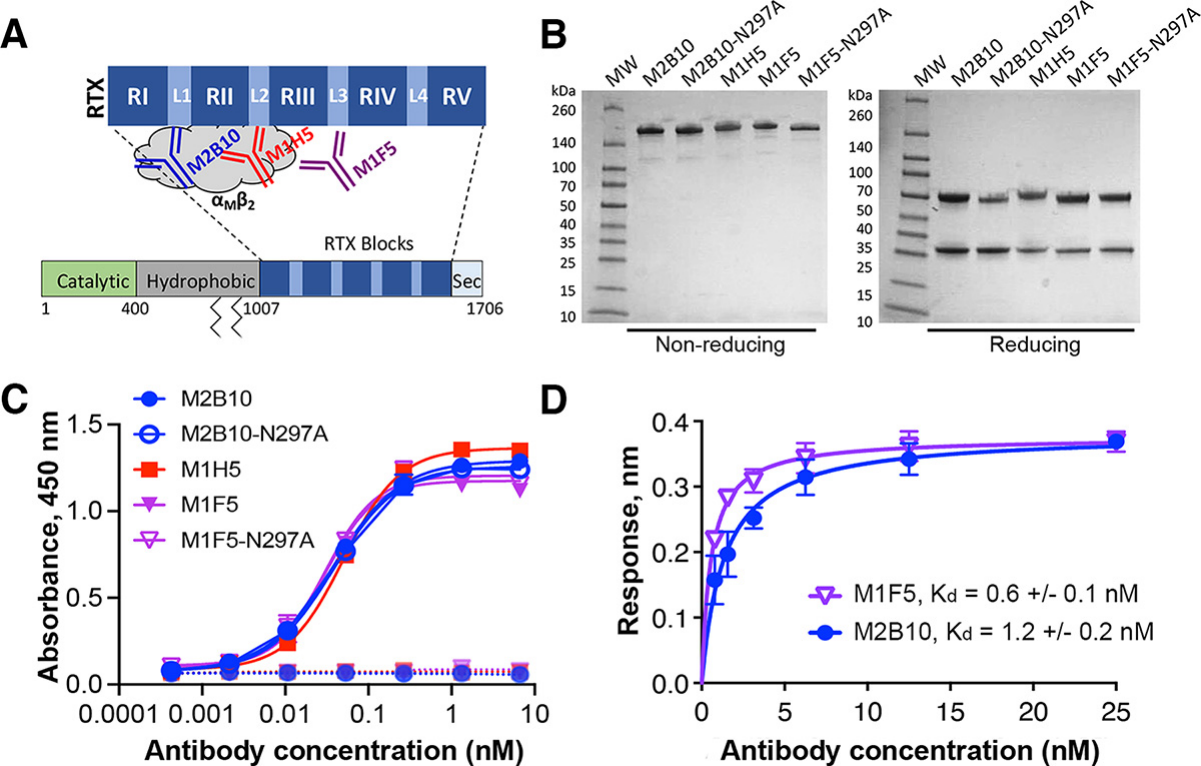
Antibodies binding different RTX epitopes have similar biophysical characteristics. (A) Schematic of the ACT domain structure showing the antibody binding sites. Mature ACT consists of a catalytically active N-terminal domain (green), a central hydrophobic domain with acylation sites at residues 860 and 983 (gray), an RTX domain comprised of five repeat blocks (RI to RV) joined by linkers (L1 to L4), and a C-terminal secretion signal (Sec). (B) SDS-PAGE gel of purified antibodies under nonreducing and reducing conditions, with molecular weight (MW) markers shown. (C) Antibody binding to wells coated with RTX_751_ (1 μg/mL) (solid lines) or uncoated wells (dashed lines) was assessed by an ELISA with anti-mouse Fc–HRP detection. The binding data were fit to a four-parameter logistic curve in GraphPad Prism. Shown are the averages from duplicates, with error bars representing the data range; the experiment was repeated twice. (D) Binding of M2B10 and M1F5 to RTX_751_ via biolayer interferometry. Antibodies were immobilized on anti-mouse Fc sensors and dipped into RTX_751_ (six concentrations from 25 to 0.8 nM) for a 30-min equilibration. Binding affinity was determined by data fitting to a Langmuir isotherm using Octet Red96 instrument software (FortéBio); the *K_d_* values are shown as the means and ranges from two replicate experiments.

Three antibodies binding the RTX domain of ACT were selected from a previously identified panel (21). Antibodies M1H5 and M2B10 neutralize ACT by sterically blocking RTX-receptor interactions, M2B10 by binding the first linker (L1) and M1H5 by binding L2 (19, 26), while the nonneutralizing antibody M1F5 binds L3 (Fig. 2A) (20). Antibody M1F5 was included to evaluate the potential for anti-ACT antibodies to directly mediate antibacterial effects such as complement lysis or opsonophagocytosis by binding to B. pertussis-associated ACT.

These antibodies were produced recombinantly as mouse IgG2a/kappa antibodies, with M2B10 and M1F5 also being expressed with an N297A substitution to impair Fc effector functions (27). These reagents exhibited high purity and typical thermostability profiles (Fig. 2B; see also Fig. S1A in the supplemental material). An enzyme-linked immunosorbent assay (ELISA) with recombinant RTX_751_ showed similar dose-response profiles for all three antibodies, suggesting that they possess similar binding affinities (Fig. 2C). Biolayer interferometry (BLI) confirmed this, revealing an M2B10 dissociation constant (*K_d_*) of 1.4 ± 0.2 nM, similar to the previously reported *K_d_* of 1.3 nM (19), and an M1F5 *K_d_* of 0.6 ± 0.1 nM (Fig. 2D; Fig. S1B). Antibody M1H5 was previously reported to have a *K_d_* of 1.6 nM (19). Since these antibodies have similar RTX affinities and biophysical characteristics, the different epitopes recognized are expected to be responsible for any functional differences.

10.1128/mbio.01527-22.1 FIG S1 Characterization of anti-RTX antibodies. (A) The thermal unfolding temperatures of anti-RTX antibodies were assessed in triplicates using a protein thermal shift dye kit (Thermo Fisher Scientific) and determined from the minimum of the transition temperatures in the derivative data [−d(fluorescence)/dT]. (B) To measure the affinity by biolayer interferometry (BLI), anti-mouse Fc sensors were coated with anti-RTX antibodies expressed with mouse constant/kappa domains to 0.4 nm, dipped into wells containing RTX_751_ at concentrations from 25 nM to 0.78 nM for 25 min, and then dipped into wells containing buffer only for a 10-min dissociation step. Equilibrium binding *K_d_* values were determined using a 1:1 Langmuir binding model with Octet analysis software. The experiment was performed twice, and representative isotherms are shown. Download FIG S1, TIF file, 0.8 MB. Copyright © 2022 DiVenere et al. 2022 DiVenere et al. https://creativecommons.org/licenses/by/4.0/ This content is distributed under the terms of the Creative Commons Attribution 4.0 International license.

### Neutralizing antibodies protect mouse and human macrophages against ACT-mediated cytotoxicity.

**FIG 3:**
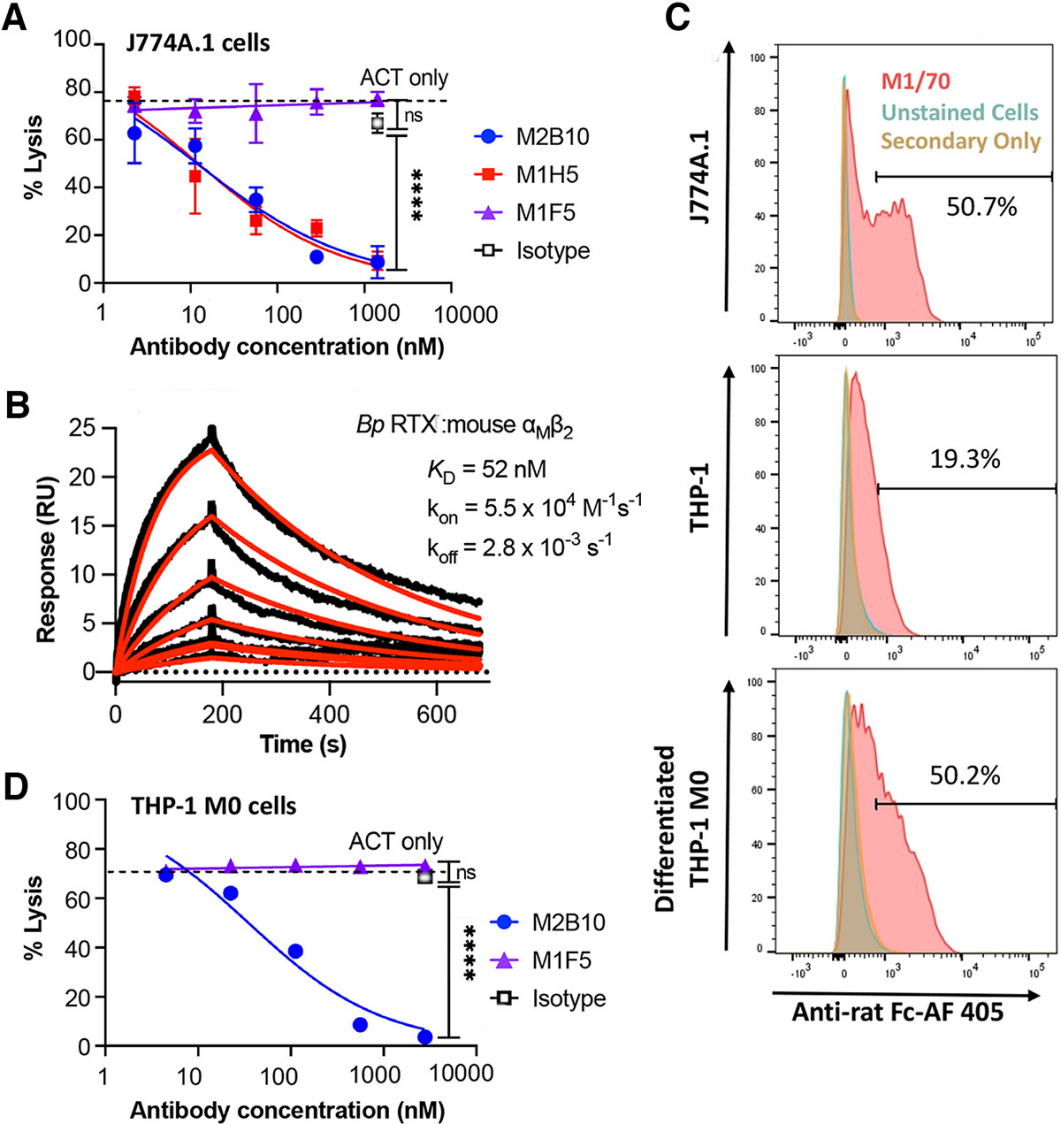
Neutralizing anti-ACT antibodies protect mouse and human macrophages against ACT cytotoxicity *in vitro*. (A) Antibody neutralization of ACT cytotoxicity was assessed with mouse macrophage J774A.1 cells. ACT (5 μg/mL) was preequilibrated with antibody (serially diluted from a 50-fold molar excess over ACT) and added to J774A.1 cells (5 × 10^5^ cells/mL) for 2 h at 37°C. Cell lysis was measured via lactate dehydrogenase release, normalized to untreated and surfactant-treated control cells to report the percentage of cell lysis. The experiment was repeated twice, with technical triplicates; error bars indicate the standard errors from duplicate assays. Reported IC_50_ values for each experiment were determined by four-parameter logistic fits in GraphPad. (B) Binding kinetics and equilibrium dissociation constants for immobilized RTX_751_ and soluble, purified mouse α_M_β_2_ integrin (2-fold dilutions from 200 to 6.1 nM) measured on a Biacore X100 instrument and analyzed using BIAevaluation software. Data are shown in black; model fits are shown in red. (C) Surface α_M_β_2_ levels on J774A.1 cells and undifferentiated and differentiated THP-1 cells were determined by flow cytometry with rat anti-α_M_β_2_ antibody M1/70 followed by anti-rat Fc–Alexa Fluor 405 (AF 405) secondary antibody. To differentiate THP-1 cells, the cells (3 × 10^5^ cells/mL) were treated with 10 ng/mL of PMA for 24 h and then allowed to rest for 72 h in PMA-free medium. The percentages of cells with the same levels of fluorescence are indicated. (D) Antibody neutralization of ACT in M0 differentiated human THP-1 cells, performed as described above for panel A but with 10 μg/mL ACT to achieve ~75% cell lysis. ns, not significant. For panels A and C, the averages and standard errors for triplicate technical replicates are shown; the experiment was repeated twice.

To better compare the levels of protection mediated by these three antibodies, we performed an *in vitro* ACT cytotoxicity assay with mouse J774A.1 macrophage cells that naturally express the murine α_M_β_2_ receptor and are commonly used to evaluate ACT activity (28). An ACT concentration in the dose-response regime was selected to readily observe antibody-mediated protection (5 μg/mL, which lysed ~75% of cells compared to the lysis control) (Fig. S2A). Preincubation of ACT with M2B10 and M1H5 reduced cell lysis in a concentration-dependent manner, with similar 50% inhibitory concentrations (IC_50_s) of 12.2 nM (±0.2 nM) and 11.8 nM (±0.7 nM), respectively. In contrast, preincubation of ACT with M1F5 at concentrations of up to 1,410 nM did not reduce cellular lysis compared to an isotype control at the same concentration or ACT alone (*P* < 0.0001 for M1F5 versus M2B10 or M1H5 at 1,410 nM) (Fig. 3A; Fig. S2B). Since M1H5 and M2B10 have similar *in vitro* ACT-neutralizing abilities but M2B10 neutralizes ACT from all *Bordetella* strains (26), it was selected for further characterization.

10.1128/mbio.01527-22.2 FIG S2 Neutralizing anti-ACT antibodies inhibit ACT cytotoxicity *in vitro*. (A) Cell lines exhibit different sensitivities to ACT toxicity. Cells were incubated with serially diluted ACT to select a concentration resulting in 75% maximum lysis for use in antibody protection assays. ACT concentrations used were 20, 10, 5, and 2.5 μg/mL for mouse J774A.1 cells and M0 differentiated and undifferentiated human THP-1 cells and 20 μg/mL for CHO-K1 cells. (B and C) Antibody-mediated *in vitro* neutralization of ACT-mediated cytotoxicity in mouse J774A.1 (B) and human THP-1 (C) cells. Antibodies were serially diluted in the presence of ACT (5 μg/mL for J774A and 10 μg/mL for THP-1 cells, starting from a 50-fold molar excess) and added to cells at 5 × 10^5^ cells/mL. After a 2-h incubation, cell lysis was measured via lactate dehydrogenase release using the CytoTox 96 kit (Promega), normalized to the values for control cells lysed with Tween, and reported as the percentage of lysis. Error bars indicate standard errors from technical triplicates; the experiment was repeated twice. Controls include untreated cells, cells treated with ACT only, and an isotype control at the highest antibody concentration. Averages and standard errors for triplicate technical replicates are shown; each experiment was repeated twice. Download FIG S2, TIF file, 0.8 MB. Copyright © 2022 DiVenere et al. 2022 DiVenere et al. https://creativecommons.org/licenses/by/4.0/ This content is distributed under the terms of the Creative Commons Attribution 4.0 International license.

To assess the relevance of the mouse model for human infection, we compared the sensitivities of human and mouse α_M_β_2_ integrins to ACT binding and intoxication. The levels of binding of ACT to each purified integrin were similar: a *K_d_* of 57 nM was previously measured by surface plasmon resonance (SPR) for RTX_751_ binding to human α_M_β_2_ integrin (20), versus 52 nM for RTX_751_ binding to mouse α_M_β_2_ integrin here, with similar on- and off-rates (*k*_on_ of 7.8 × 10^4^ versus 5.5 × 10^4^ M^−1^ s^−1^ and *k*_off_ of 4.4 × 10^−3^ versus 2.8 × 10^−3^ s^−1^ for human versus mouse integrins, respectively) (Fig. 3B). To assess ACT intoxication of human monocytic THP-1 cells, these cells were first differentiated to adherent M0 (non-activated) macrophages as this process upregulates the human α_M_β_2_ receptor (29), which was verified by staining with a rat anti-α_M_β_2_ antibody and flow cytometry (Fig. 3C). As expected, THP-1 differentiation conferred increased ACT sensitivity due to the higher surface receptor density (Fig. S2C). When using THP-1 M0 cells, 10 μg/mL ACT reproducibly lysed ~75% of cells (Fig. S2A), and this concentration was selected for use in neutralization assays. Antibody protection of THP-1 M0 cells showed trends similar to those for J774A.1 cells, with higher IC_50_ values due to the higher ACT concentration used: M2B10 had an IC_50_ of 40.4 nM (±5.1 nM), while M1F5 again showed no change in cells lysis versus the isotype control at 2,800 nM (Fig. 3D; Fig. S2C). Despite naturally infecting only humans, these data show that B. pertussis RTX_751_ binds purified α_M_β_2_ integrins and that ACT intoxicates macrophages from humans and mice similarly. Overall, these results suggest that ACT neutralization during experimental mouse infection will provide data relevant for human infection.

### Antibody delivery by nasal administration results in high lung bioavailability.

ACT is secreted from B. pertussis, and while some ACT may associate with filamentous hemagglutinin (Fha) at the bacterial surface (30), ACT also intoxicates nearby phagocytes (22). Unlike the pertussis toxin, which exerts systemic effects and can be potently neutralized by systemically administered antibodies (31, 32), ACT activities are thought to be restricted to the respiratory environment (22). We therefore hypothesized that anti-ACT antibodies would need to be present at the mucosal surface to effectively find, bind, and block ACT activities. We accordingly compared antibody delivery to mice by three administration routes to identify a method resulting in high lung bioavailability.

We initially compared antibody delivery to the lungs by nasal droplets and a nebulizer using the model antibody hu1B7 to supply the quantities required for nebulization and because it shares many biophysical characteristics with M2B10 and M1F5. Protein activity was not impacted by aerosolization, as assessed by an SDS-PAGE protein gel and an antigen binding ELISA (Fig. S3). Antibody (46 μg) or phosphate-buffered saline (PBS) was administered to mice by aerosol or nasal drip, with lung antibody concentrations being measured 17 h later: hu1B7 antibody was undetectable in PBS-treated mice, whereas those receiving antibody by nasal drip had 7.66 ± 4.42 μg/mL and those receiving antibody by a nebulizer had 0.026 ± 0.018 μg/mL hu1B7 (*P* < 0.01) (Fig. S4A). Higher antibody lung bioavailability after delivery by nasal drip than by aerosol delivery has been reported previously (33). Since the nasal drip method requires far less protein, is technically straightforward, and achieves higher lung antibody concentrations, this approach was used in subsequent experiments.

10.1128/mbio.01527-22.3 FIG S3 hu1B7 antibody integrity and antigen binding activity after aerosolization. (A) A 12% SDS-PAGE gel was used to compare nonaerosolized control hu1B7 antibody with aerosolized antibody (“A”) and antibody recovered from the nebulizer reservoir (“R”) under nonreducing and reducing conditions. Samples shown are from three replicate experiments (indicated by 1, 2, and 3). (B) An ELISA was used to compare the antigen binding activities of control hu1B7 antibody with aerosolized antibody samples and coated pertussis toxin (PTx) antigen. Shown are the averages and standard deviations from two technical replicates from a single nebulizer experiment as well as four-parameter logistic fits generated using GraphPad Prism 5 (lines). The data shown are from one of three repeated experiments (experiment 1 shown in panel A) and are representative of results from all three experiments. Download FIG S3, TIF file, 0.8 MB. Copyright © 2022 DiVenere et al. 2022 DiVenere et al. https://creativecommons.org/licenses/by/4.0/ This content is distributed under the terms of the Creative Commons Attribution 4.0 International license.

10.1128/mbio.01527-22.4 FIG S4 Antibody lung bioavailabilities compared by delivery method. (A) The concentrations of hu1B7 antibody in homogenized lung samples from mice receiving antibody by intranasal drip or aerosol delivery, or PBS only by intranasal drip as a control, were analyzed by an ELISA. The antibody drip method delivered significantly more antibody than the aerosol method (**, *P* < 0.01 [by ANOVA with Tukey’s posttest for multiple comparisons]). (B) The M2B10 antibody concentrations in sera and homogenized lung samples from mice (*n* = 3) administered antibody through an intranasal (i.n.) drip or intraperitoneal (i.p.) injection were quantified by an ELISA (insignificant [lungs, *P* = 0.0943; sera, *P* = 0.0961 {by a paired *t* test}]). Download FIG S4, TIF file, 0.8 MB. Copyright © 2022 DiVenere et al. 2022 DiVenere et al. https://creativecommons.org/licenses/by/4.0/ This content is distributed under the terms of the Creative Commons Attribution 4.0 International license.

We next compared lung bioavailabilities after intraperitoneal (i.p.) injection versus nasal droplet administration. Since these experiments require modest amounts of antibody, we used the ACT-neutralizing antibody M2B10 with mouse IgG2a/kappa constant domains to support Fc receptor-mediated functions, including transport between the sera and mucosal tissues. Antibody injection resulted in nearly 10-fold-higher serum concentrations than nasal drip, while intranasal (i.n.) administration resulted in nearly 10-fold-higher lung concentrations 17 h after administration (Fig. S4B). These trends are consistent with those reported in previous studies (34, 35) and support antibody administration by nasal delivery to achieve high antibody lung bioavailability. Pulmonary delivery is increasingly considered as a strategy to deliver antibody therapeutics for respiratory pathogens, including Pseudomonas aeruginosa (36) and respiratory syncytial virus (37), since the transport of IgG from the sera to mucosal surfaces is inefficient.

### Antibodies neutralizing ACT protect against lethal B. pertussis infection by receptor blockade.

**FIG 4:**
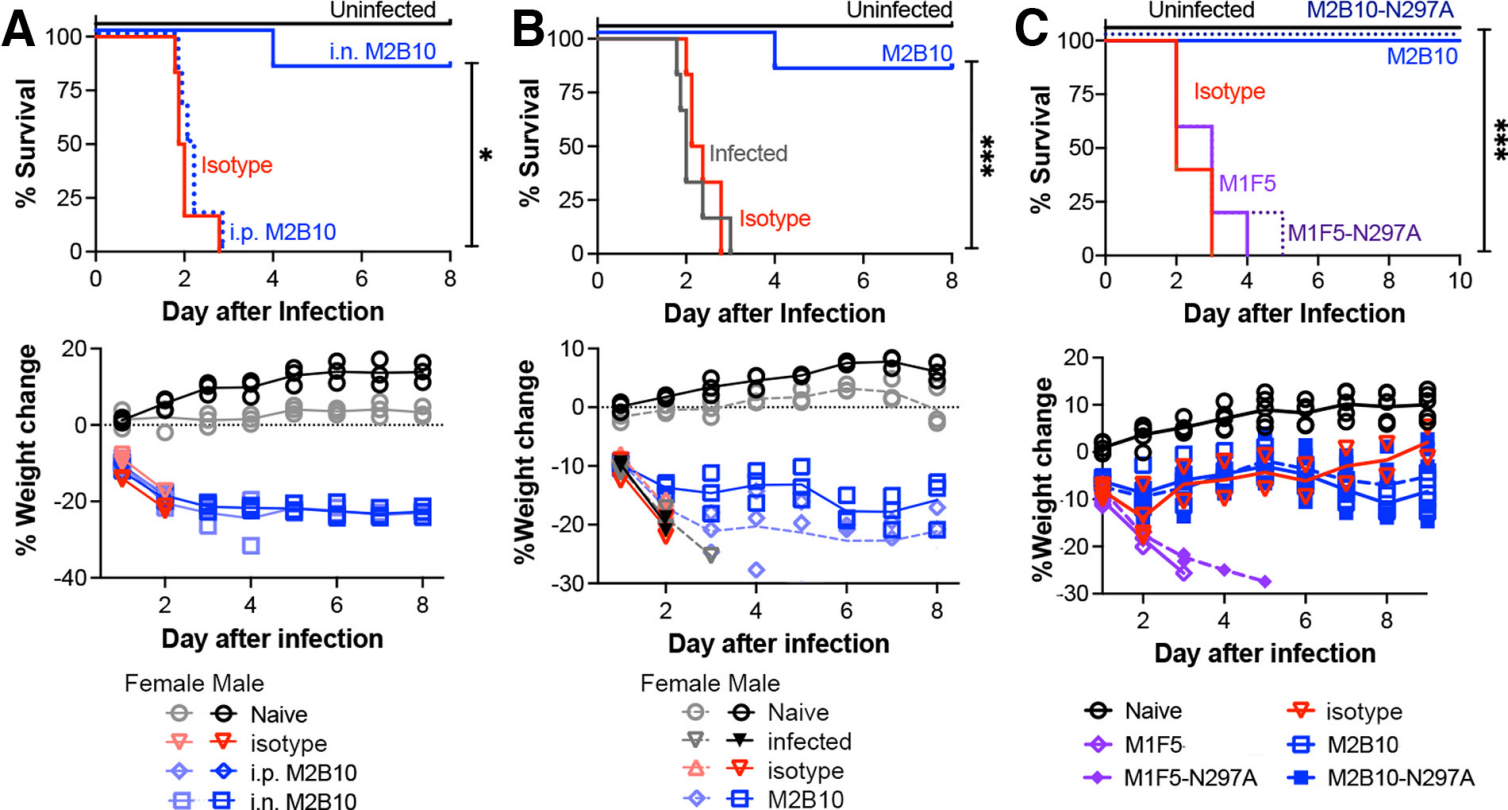
Intranasal administration of ACT-neutralizing antibody protects mice against lethal pertussis challenge. (A) BALB/c mice (*n* = 6 per group; half female; 32 days old) were administered 10 μg M2B10 or an isotype control antibody intranasally (i.n.) or M2B10 intraperitoneally (i.p.) 2 h prior to challenge with a high dose (2 × 10^8^ CFU) of B. pertussis TohamaI. All antibodies were produced with mouse IgG2a/kappa constant domains. (B) Five micrograms of M2B10, 10 μg of the isotype control, or the PBS control was administered intranasally to 32-day-old BALB/c mice (*n* = 6; half female) before infection with 2 × 10^8^ CFU B. pertussis TohamaI. (C) Antibodies M2B10 and M1F5 and their aglycosylated N297A counterparts (10 μg M2B10, 10 μg M2B10-N297A, 20 μg M1F5, and 20 μg M1F5-N297A) were administered intranasally to mice (*n* = 5) before infection with 2 × 10^8^ CFU B. pertussis TohamaI. For all panels, *** indicates a *P* value of <0.001 as determined by the Mantel-Cox test with a Bonferroni-Dunn posttest for multiple comparisons.

As an initial evaluation of the potential for anti-ACT antibodies to protect mice against lethal pertussis infection, we administered 10 μg of the neutralizing antibody M2B10 by intraperitoneal injection 2 h before infection or by nasal droplets shortly before lethal infection with 2 × 10^8^ CFU B. pertussis TohamaI. While all uninfected mice remained healthy, all mice administered the control antibody and those receiving M2B10 by intraperitoneal injection succumbed within 3 days. This is similar to previous reports in which mouse infection with 1 × 10^8^ CFU B. pertussis TohamaI resulted in complete lethality in 2 days (10, 38). In contrast, just one of six mice receiving M2B10 intranasally succumbed to infection. Mouse weight changes provided a second metric of overall health and were a key component of the morbidity criteria (Fig. 4A). This experiment was repeated with a lower dose of intranasally administered M2B10 (5 μg), with similar results (Fig. 4B). Both experiments used male and female mice, with an impact of gender on survival being detected with these small group sizes (*n* = 3 per gender per treatment) (Fig. S5). These data support the need for high anti-ACT concentrations in the respiratory tract to observe protective effects.

10.1128/mbio.01527-22.5 FIG S5 Gender effect on the mouse model of infection. BALB/c mice were infected with 2 × 10^8^ CFU B. pertussis TohamaI (*n* = 30 mice per experiment; 5 groups, each with 3 males and 3 females). Means reflect outcomes for infected mice from two similar experiments performed on different weeks. n.s., not significant (by an unpaired *t* test). Download FIG S5, TIF file, 0.8 MB. Copyright © 2022 DiVenere et al. 2022 DiVenere et al. https://creativecommons.org/licenses/by/4.0/ This content is distributed under the terms of the Creative Commons Attribution 4.0 International license.

To better understand how anti-ACT antibodies contribute to protection against lethal pertussis challenge, we compared the levels of protection by neutralizing M2B10, nonneutralizing M1F5, and their Fc-silenced N297A counterparts. Mice were again administered antibody intranasally, followed by 2 × 10^8^ CFU B. pertussis TohamaI. All mice receiving the nonneutralizing M1F5 and M1F5-N297A antibodies met euthanasia criteria by days 4 to 5, while all naive mice and those receiving the neutralizing M2B10 and M2B10-N297A antibodies survived (Fig. 4C). These data indicate that a simple blockade of ACT-receptor interactions is sufficient to protect mice from pertussis without requiring Fc effector functions.

### ACT-neutralizing antibodies synergize with antipertactin antibodies to reduce lung bacterial colonization.

**FIG 5:**
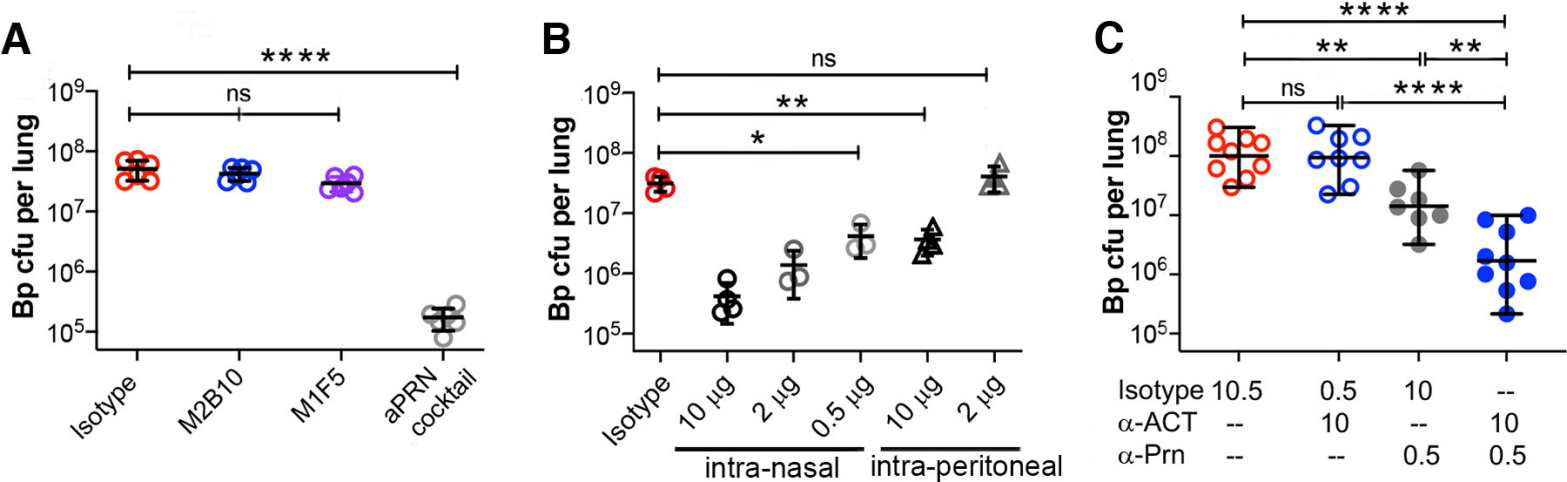
Anti-ACT antibodies synergize with antipertactin antibodies to decrease lung bacterial colonization. Using a pneumonia colonization model, mice were administered antibody intranasally and then infected with a sublethal dose of B. pertussis TohamaI (5 × 10^6^ CFU) before harvesting the lungs on day 3 after infection to enumerate B. pertussis CFU. (A) Mice (*n* = 5) were intranasally administered 10 μg of a single anti-ACT antibody or a cocktail of antipertactin (aPRN) antibodies as a positive control to reduce colonization. (B) Mice (*n* = 4) were administered different amounts of the antipertactin antibody 2E9 via intranasal or intraperitoneal administration to determine a partially protective dose. Data shown are from the same experiment; isotype and intranasal data were previously reported (39). (C) To evaluate potential synergies between the antipertactin 2E9 and neutralizing anti-ACT M2B10 responses, mice were intranasally administered a partially protective 2E9 dose (0.5 μg), an M2B10 dose (10 μg), and/or an isotype control antibody for a total antibody dose of 10.5 μg. Data are pooled from two experiments, each with 3 to 5 mice. For all panels, * indicates a *P* value of <0.05, ** indicates a *P* value of <0.01, and **** indicates a *P* value of <0.0001 as determined by one-way analysis of variance (ANOVA) and Tukey’s *post hoc* test to determine *P* values between groups.

To determine whether anti-ACT antibodies reduce B. pertussis lung bacterial colonization levels during the early stages of infection, we infected mice with a lower bacterial dose (5 × 10^6^ CFU) and measured lung B. pertussis levels on day 3 after infection. Individual anti-ACT antibodies, a cocktail of four antipertactin antibodies (39), or controls were administered to mice intranasally before B. pertussis infection. Mice treated with only the isotype control antibody, M2B10, or M1F5 had similar bacterial colonization levels of ~10^7^ CFU per mouse lung, whereas treatment with an antipertactin cocktail reduced lung levels ~100-fold (*P* < 0.0001) (Fig. 5A). These data indicate that anti-ACT antibodies, while protective against lethal pertussis challenge, do not significantly reduce lung bacterial colonization levels on their own, even when present at high concentrations in the lungs.

Previous reports suggested that anti-ACT neutralization enhanced protection by immune sera *in vitro* (40, 41), while pertactin immunization has been shown to induce bactericidal responses (42). Accordingly, we hypothesized that neutralizing anti-ACT antibodies may synergize with antipertactin antibodies *in vivo* by protecting phagocytes, which can then more effectively target opsonized bacteria. To evaluate this possibility, we determined a dose and a route of administration of the bactericidal antipertactin antibody 2E9 (39) that provided a low but significant reduction in lung colonization. The intranasal administration of 10, 2, or 0.5 μg 2E9 correlated with lung colonization levels (Pearson correlation coefficient of −0.93), with even 0.5 μg significantly reducing B. pertussis colonization compared to isotype control-treated mice (~10-fold [*P* < 0.001]) (Fig. 5B). Interestingly, the intraperitoneal administration of 10 μg 2E9 resulted in the same colonization level as that in mice receiving 0.5 μg intranasally. In addition to identifying 0.5 μg as a minimally protective intranasal 2E9 dose for use in synergy studies, these data support the benefits of mucosally available antipertactin antibodies.

We next evaluated whether anti-ACT and antipertactin antibodies synergize to limit B. pertussis lung colonization. Mice were intranasally administered a high dose of M2B10 (10 μg) and a low dose of 2E9 (0.5 μg), alone or in combination, before infection with 5 × 10^6^ CFU B. pertussis TohamaI (Fig. 5C). All treatment groups were administered the same total amount of antibody (10.5 μg) by varying the dose of the isotype control. Whereas mice treated with the isotype control or high-dose M2B10 with the isotype control had identical high lung colonization levels (~10^8^ CFU) and mice treated with a low dose of 2E9 had ~10-fold-reduced colonization levels (~10^7^ CFU [*P* < 0.01]), mice treated with the M2B10-2E9 cocktail showed clear evidence of synergistic protection, which reduced colonization levels an additional 10-fold to ~10^6^ CFU (*P* < 0.0001). Whereas additive effects between anti-ACT and antipertactin would reduce colonization 10-fold, synergies between these two antibodies led to a 100-fold reduction. These data support the hypothesis that anti-ACT and antipertactin antibodies act synergistically to reduce bacterial colonization during *in vivo* infection.

## DISCUSSION

While immunization with acellular pertussis vaccines succeeds in preventing disease symptoms and protecting most infants, it has not disrupted bacterial transmission. A study of 18 European countries with high vaccination coverage estimated an annual infection risk of ~2 to 5% per person (43), while studies in Africa suggest that the pertussis incidence is underreported (44). Accordingly, there is growing interest in the development of next-generation pertussis vaccines and a corresponding need to understand the mechanisms by which key virulence factors can contribute to protection. ACT is a promising vaccine candidate, but its unclear role in protection (16) as well as challenges in protein production (45, 46) precluded its inclusion in current acellular vaccine formulations. Here, we used recombinant antibodies binding structurally defined epitopes to demonstrate the role of ACT-neutralizing antibodies in protection. Our results reconcile previous conflicting data to provide a unifying interpretation of ACT-mediated protection against infection.

Previous efforts to evaluate ACT’s role in protection used primarily immunization with full-length ACT alone or in combination with acellular pertussis vaccines, followed by mouse infection. These studies reported a modest adjuvant effect that elevated antipertactin IgG and IgA responses (15), which could not be uncoupled from the possible contributions of anti-ACT antibodies to protection via neutralization, opsonization, or another mechanism. Moreover, the anti-ACT response after immunization was likely suboptimal for several reasons. First, ACT is a large, complex protein that readily aggregates, especially in its unacylated form (46). ACT immunization with the posttranslational acylation required for proper folding conferred stronger adjuvant effects than unacylated ACT, suggesting that proper folding is key to eliciting protective effects, whereas the adenylate cyclase activity was not required (15, 47, 48). Immunization with ACT prepared by standard refolding methods elicits many nonneutralizing antibodies (21, 49), further suggesting that only a fraction of the ACT epitopes elicit neutralizing responses. Second, these efforts used primarily parenteral immunization as opposed to strategies designed to elicit strong mucosal responses. Since ACT acts in proximity to the producing bacteria with no known systemic effects, colocalization of anti-ACT antibodies and bacteria may be required to intercept newly secreted ACT molecules *en route* to a target cell. Third, ACT produced during an infection impairs innate immune responses by potently inhibiting α_M_β_2_ and Fc receptor-mediated phagocytosis and related antibacterial activities mediated by neutrophils and macrophages (40, 50, 51). Thus, ACT neutralization may primarily serve to augment antipertussis immune responses, but neutralizing mucosal antibodies are poorly elicited by parenteral immunization with refolded ACT.

To address these shortcomings and to uncouple ACT contributions to protection, we first administered monoclonal anti-ACT antibodies to naive mice before experimental infection. A low dose of the neutralizing antibody M2B10 protected against lethal pertussis challenge, but neither the nonneutralizing antibody M1F5 nor an isotype control had any effect (Fig. 4A and B). Since an Fc-silenced version of M2B10, M2B10-N297A, was also protective (Fig. 4C), we conclude that protection was achieved by the simple blockade of ACT-receptor interactions as the antibodies were otherwise biochemically similar (Fig. 2 and 3; see also Fig. S1 in the supplemental material). In the naive mouse, ACT neutralization could support bacterial elimination via the alternative complement pathway (52, 53). This pathway can be triggered by pertussis lipooligosaccharides to deposit C3b on the bacterial surface, which, after conversion to iC3b, binds the α_M_β_2_ receptor to trigger phagocytosis (54, 55). *In vitro*, large amounts of naive serum efficiently kill wild-type B. pertussis (39, 56, 57), with different isolates exhibiting various sensitivities based on the recruitment of complement inhibitors (42, 55). Since B. pertussis employs strategies to evade complement, and B. pertussis phagocytosis via α_M_β_2_ is generally less efficient than Fc-mediated phagocytosis (52), the overall impact may be modest but sufficient to allow mouse survival after a high-dose bacterial infection, which is less impacted by complement evasion (58).

Interestingly, whereas mouse infection with a B. pertussis strain producing catalytically inactive ACT showed ~10-fold-lower lung colonization levels on day 3 than infection with a wild-type strain (Fig. 1), treatment with the M2B10 ACT-neutralizing antibody alone did not reduce colonization (Fig. 5A). Multiple previous reports (9, 25, 50, 51) have observed similar colonization defects after infection of naive mice with ACT-impaired B. pertussis strains, suggesting that treatment with neutralizing anti-ACT antibodies, whether elicited via passive or active immunization, could achieve at most a 10-fold reduction in colonization. Antibody access to the site of ACT activity in the respiratory tract appears to limit the level of protection observed since intranasal, but not systemic, antibody administration conferred protection (Fig. 4A). Antibody access to ACT may be further restricted: the failure of exogenous ACT to complement the ΔACT phenotype in mice after nasal delivery (25) is consistent with the idea that ACT activity may require secretion adjacent to a leukocyte membrane and/or that ACT can intoxicate leukocytes after bacterial internalization, as has been reported previously for Staphylococcus aureus leukotoxin AB (59). The administration of multiple antibody doses or cocktails of ACT-neutralizing antibodies binding different epitopes (e.g., M2B10 and M1H5) may allow antibody treatment to access these sites and fully recapitulate the ACT deletion phenotype.

Hypothesizing that the preservation of phagocyte activities may be most useful in the presence of opsonizing antibodies, we evaluated the potential for ACT neutralization to synergize with a bactericidal antipertactin monoclonal antibody (39). Previous *in vitro* assays with human neutrophils showed that monoclonal antibodies neutralizing ACT contribute to B. pertussis killing only in the presence of opsonizing human immune sera (40). We similarly observed that anti-ACT antibodies exhibit synergistic effects *in vivo* when combined with opsonizing antibodies that rely on phagocytic cells: lung colonization levels were reduced ~10-fold more than would be expected from the additive effects of each antibody alone (Fig. 5C). This colonization level is comparable to the maximum decrease observed after treatment with high doses of antipertactin antibodies (Fig. 5A and B). This experiment mimics protection in an immune host, in which ACT-sensitive, Fc-dependent antibody-mediated phagocytosis by neutrophils plays a key role (60). While pertactin is a less relevant target due to the widespread emergence of pertactin-negative strains, the synergy observed likely applies to other highly expressed surface proteins under consideration as vaccine antigens, such as Vag8 and BrkA (61, 62).

B. pertussis colonizes the upper and lower respiratory tracts, and there is a growing consensus that immune responses in these tissues will be crucial to protect against disease and reduce transmission (63). Notably, protection by anti-ACT antibodies in this work was observed after direct administration to the respiratory tract intranasal but not systemic administration (Fig. 4A); the intranasal delivery route also greatly reduced the dosage of antipertactin antibody required to reduce lung bacterial colonization (Fig. 5B). Intranasal delivery coats the upper respiratory tract with antibody before delivering some of the inoculum to the lungs (Fig. S4) and thus has the potential to also reduce colonization in the nasal tissues. However, the key limitations of this study are that we did not measure bacterial colonization in the trachea and nose, nor did we evaluate the impact of antibody treatment on bacterial transmission.

Next-generation pertussis vaccine strategies include the addition of new antigens and adjuvants to existing acellular formulations as well as novel vaccine formulations with more complex antigen mixtures. The results shown here highlight the need for vaccine designs that elicit strong antibody responses in the respiratory tract and the potential for neutralizing anti-ACT responses to synergize with opsonizing antibodies binding other pertussis targets. Intranasal vaccination with appropriate adjuvants can induce strong and durable immune responses in these tissues (64) and may elicit the antibody levels achieved here through passive administration. Accordingly, future work will evaluate the potential for mucosal vaccination with engineered ACT immunogens to focus immune responses on neutralizing epitopes (19) and, when combined with opsonizing antigens, diminish B. pertussis colonization in the upper and lower respiratory tracts.

## MATERIALS AND METHODS

### Antibody production and characterization.

The variable light and heavy domains of anti-ACT antibodies M2B10, M1H5, and M1F5 (21) and an isotype control that is specific for a human cytomegalovirus epitope (65) or each of these antibodies was introduced into Abvec plasmids via AgeI and BsiWI or AgeI and SalI cut sites (66), respectively, upstream of an in-frame mouse Ig kappa or IgG2a hinge and Fc domain. Antibodies were produced in ExpiCHO-S cells (Gibco) according to the manufacturer’s high-titer protocol. ExpiCHO-S cells were grown to 7 × 10^6^ to 10 × 10^6^ cells/mL in 25 mL ExpiCHO expression medium at 37°C with 8% CO_2_ at 125 rpm. On the day of transfection, cells were diluted to 6 × 10^6^ cells/mL in fresh medium and transfected with 25 μg DNA complexed with ExpiFectamine in OptiPRO, according to the manufacturer’s instructions. After transfection, ExpiCHO cells were grown at 37°C with 8% CO_2_ for 20 h, after which ExpiCHO feed and ExpiCHO enhancer were added, and cultures were moved to 32°C with 5% CO_2_ for 10 to 12 days of expression. Antibodies were purified from the culture supernatant using a 1-mL HiTrap protein A column on an Äkta fast protein liquid chromatography (FPLC) system (GE Healthcare) according to the manufacturer’s instructions. The column was washed with a solution containing 25 mM Tris and 25 mM NaCl (pH 7.4) and eluted with a solution containing 100 mM sodium citrate and 50 mM NaCl (pH 3). Eluted fractions were pooled, buffer exchanged into phosphate-buffered saline (PBS) (10-kDa-molecular-weight cutoff [MWCO]; Amicon), and stored at 4°C until use.

### Antigen production and characterization.

Acylated ACT was produced as inclusion bodies and refolded as described previously (21). The RTX domain, encoding residues 751 to 1706 of ACT, with the addition of a single N-terminal Strep tag (IBA), was introduced into the NheI and BamHI restriction sites of plasmid pET28a+ and expressed in the cytoplasm of Escherichia coli BL21(DE3) cells. A bacterial culture grown overnight in TB medium with 1% glucose and 50 μg/mL kanamycin was diluted 1:100 into fresh TB medium with 50 μg/mL kanamycin and grown at 37°C at 225 rpm until the optical density at 600 nm (OD_600_) reached ~0.6. The culture was chilled on ice for 15 min to reduce the temperature before induction with 0.4 mM isopropyl-β-d-thiogalactopyranoside (IPTG) for ~5 h at 21°C at 225 rpm. Cells were collected by centrifugation, resuspended in an ~1/20 culture volume of HBSC (HEPES-buffered saline [HBS] plus 2 mM CaCl_2_), and lysed on ice with a sonicator (catalog number S-125-110; Qsonica) using 75% power and 6 cycles of 10 s on and 20 s off. The lysate was clarified by centrifugation at 20,000 rpm at 4°C for 20 min, filtered with a 0.22-μm filter, and applied directly to a 1-mL StrepTactin-XT Superflow high-capacity column (catalog number 2-4025-001; IBA) on an Äkta FPLC system. The column was washed with HBSC, and bound protein was eluted with HBSC plus 50 mM biotin (Sigma). Fractions were pooled, dialyzed into HBSC with Slide-A-Lyzer cartridges (Thermo Scientific), quantified by a bicinchoninic acid (BCA) assay (Pierce), aliquoted, and frozen at −80°C until use.

### Integrin production and characterization.

A gene encoding mouse α_M_ residues 1 to 1105 was cloned into pαH with a C-terminal disulfide-bond-forming linker (GCGG) (67), an HRV 3C cleavage site (LEVLFQGP), an acid coiled-coil heterodimerization motif (GENAQCEKELQALEKENAQLEWELQALEKELAQ) (68), and two Strep-tag II tags separated by a Gly-Ser linker. A gene encoding β_2_ residues 1 to 701 was cloned into pαH with a C-terminal disulfide-bond-forming linker (DGCG) (67), an HRV 3C cleavage site (LEVLFQGP), a base coiled-coil heterodimerization motif (GKNAQCKKKLQALKKKNAQLKWKLQALKKKLAQGG) (68), and a 6×His tag. The plasmids encoding the mouse α_M_ ectodomain and the mouse β_2_ ectodomain were cotransfected into FreeStyle 293-F cells (Invitrogen) for 6-day expression. The cell supernatant was harvested and passed through a 0.22-μm filter, which was then buffer exchanged into a solution containing 20 mM Tris (pH 7.5), 150 mM NaCl, 1 mM CaCl_2_, and 1 mM MgCl_2_ using tangential flow filtration. The integrin heterodimer was purified from the buffer-exchanged supernatant using StrepTactin XT resin equilibrated with a solution containing 20 mM Tris (pH 7.5), 150 mM NaCl, 1 mM CaCl_2_, and 1 mM MgCl_2_. Resin was washed with a solution containing 20 mM Tris (pH 7.5), 150 mM NaCl, 1 mM CaCl_2_, and 1 mM MgCl_2_, and elution was performed with a solution containing 20 mM Tris (pH 7.5), 150 mM NaCl, 1 mM CaCl_2_, 1 mM MgCl_2_, and 50 mM biotin. Eluted protein was further purified by running it over a Superdex 200 10/300 gel filtration chromatography column (Cytiva).

### Enzyme-linked immunosorbent assays.

Enzyme-linked immunosorbent assays (ELISAs) were performed to assess antigen and antibody conformations and functional integrity. Briefly, 96-well adhesive plates (Corning) were coated with RTX or pertussis toxin (List Labs) at 0.5 μg/mL in HEPES-buffered saline with 10 mM calcium (HBSC) or 0.2 μg/mL in PBS, respectively, overnight at 4°C. Plates were then washed once with PBS plus 0.05% Tween 20 (PBS-T) and blocked with a double coat volume of PBS-T with 5% milk for 1 h at room temperature. Purified antibody was added in duplicate wells to the top row of the plate, diluted 1:5 from a 2-μg/mL starting concentration, and incubated at room temperature for an hour. Plates were washed three times with PBS-T before the addition of a 1:2,000 dilution of anti-mouse Ig–horseradish peroxidase (HRP) (Santa Cruz) or anti-human-kappa–HRP (SouthernBiotech) for 1 h. Plates were then washed three times, the signal was developed with the tetramethylbenzidine (TMB) substrate (Pierce) and quenched with 0.1 N HCl, and the absorbance at 450 nm was measured with a SpectraMax M5 plate reader. Data were exported for analysis in GraphPad Prism 8.

To determine antibody concentrations in serum and other samples, ELISAs were performed as described above with a purified antibody of a known concentration plated in a duplicate dilution series using PBS-T–milk as the diluent. Samples of unknown concentrations were similarly titrated in duplicate, and plates were processed as described above. Data were analyzed by first correlating the purified antibody concentration and absorbance values in GraphPad using the four-parameter logistic equation *y* = {(*A* − *D*)/[1 + (*x*/*C*)*^B^*]} + *D*. The unknown samples were then fit using these four parameters (*A*, *B*, *C*, and *D*), and the unknown concentration, *x*, was adjusted to minimize the error between the calculated and observed absorbances, *y*, using Solver in Excel. The concentration was then adjusted for the dilution factor used in the ELISA to determine the concentration in the sample, and the concentrations calculated for each replicate sample dilution series were averaged.

### Measurement of protein-protein binding kinetics.

To determine the equilibrium binding affinities of anti-ACT antibodies for RTX using BLI, anti-mouse IgG Fc (AMC; FortéBio) sensors were loaded with antibodies at 10 nM in kinetic buffer (0.01% bovine serum albumin [BSA], 0.002% Tween 20, and 2 mM CaCl_2_ in PBS) to a response of 0.4 nm. Association curves with RTX at six concentrations from 50 nM to 0.8 nM in 2-fold dilution steps were recorded for 25 min. The dissociation was recorded for 10 min after tip transfer to fresh kinetic buffer. Steady-state *K_d_* values were determined by Langmuir isotherm analysis using Octet Red96 instrument software (FortéBio).

To measure the binding kinetics of RTX for purified mouse α_M_β_2_ integrin using a Biacore X100 system, the M1F5 Fab was desalted into 10 mM Na-acetate (pH 4.0) and coupled onto both flow cells to ~2,500 response units (RU) of a CM5 SPR biosensor (catalog number BR100012; Cytiva) using an EDC/NHS amine coupling kit (catalog number BR100050; Cytiva). The running buffer used for kinetic measurements contained 10 mM HEPES (pH 7.4), 150 mM NaCl, 1 mM CaCl_2_, 1 mM MgCl_2_, and 0.05% Tween 20. For each cycle of α_M_β_2_ binding, ~10 RU RTX (10 nM) (5-μL/min flow rate for 15 s) was coupled to flow cell 2. Subsequently, buffer (for double reference subtraction) or mouse α_M_β_2_ at 6.13 nM, 12.5 nM, 25 nM, 50 nM, 100 nM, or 200 nM was flowed into both flow cells for 180 s to measure association. Running buffer was subsequently flowed in for 600 s to measure dissociation. Data were fit to a global 1:1 binding model for the determination of kinetic parameters using BIAevaluation software.

### Toxin neutralization assay.

Mouse macrophage-like J774A.1 cells (ATCC TIB-67) or human monocyte THP-1 cells (ATCC TIB-202) were grown in Dulbecco’s modified Eagle’s medium (DMEM) supplemented with 10% fetal bovine serum (FBS), 1 mM sodium pyruvate, and penicillin-streptomycin (Sigma) or RPMI 1640 supplemented with 10% FBS and penicillin-streptomycin, respectively. J774A.1 cells were scraped using a rubber spatula before subculturing or seeding for assays. Human monocytic THP-1 cells were differentiated to the M0 macrophage phenotype to cause an increased expression level of the α_M_β_2_ receptor for *in vitro* assays. THP-1 cells (300,000 cells/mL) in antibiotic-free complete RPMI 1640 medium were treated with 10 ng/mL of phorbol 12-myristate 13-acetate (PMA) for 24 h. After 24 h, nonadherent cells were aspirated with the PMA medium and replaced with fresh PMA-free medium for 72 more hours (29). Treatment of cells with PMA causes a change in morphology from suspension cells to adherent cells. To detect the upregulation of α_M_β_2_, cells were incubated with rat anti-α_M_β_2_ antibody M1/70 (BioLegend) at 10 μg/mL for 45 min on ice and detected with the secondary antibody anti-rat Ig–Dylight-405 (Jackson ImmunoResearch) at 1:2,000 using a BD Fortessa flow cytometer.

One day prior to performing an ACT toxin neutralization assay with J774A.1 cells, 1 × 10^5^ J774A.1 cells were seeded per well in 200 μL medium into a 96-well tissue culture plate (catalog number 07-200-87; Fisher). PMA-differentiated THP-1 cells were seeded at 1 × 10^5^ cells per 200 μL directly before each assay. Wells were seeded for triplicate technical replicates for samples and assay controls (untreated cells to monitor spontaneous lysis and surfactant-treated cells to monitor maximum lysis). To perform the assay, an ACT stock solution in 8 M urea was rapidly diluted to a 2× working concentration of 10 μg/mL or 20 μg/mL in unsupplemented DMEM or RPMI 1640, respectively, and vortexed briefly. Since the refolding process is sensitive to small protocol changes and the refolded ACT is prone to aggregation (46), the stock solution was prepared immediately before each experiment, and the same stock was used for all ACT-treated wells in the assay. One hundred eighty microliters of the refolded ACT stock was added to one Eppendorf tube per assay condition containing 180 μL of unsupplemented DMEM or RPMI 1640 with antibodies starting at a 50-fold molar excess of ACT or 5-fold serial dilutions of the antibodies incubated for 30 min.

During this incubation step, cells were washed three times with prewarmed PBS and aspirated to remove wash buffer. ACT-antibody complexes (110 μL per well) were added to triplicate wells, or unsupplemented DMEM or RPMI 1640 (110 μL per well) for the untreated control wells, and 100 μL of DMEM or RPMI 1640 was added to the lysis control wells. The plate was incubated at 37°C with 5% CO_2_ for 75 min. At this time, 10 μL of lysis reagent (9% [vol/vol] Triton X-100) was added to the lysis control wells, and the plate was incubated for an additional 45 min at 37°C with 5% CO_2_. To detect lactate dehydrogenase released from lysed cells, the supernatant (40 μL) was transferred to an untreated 96-well assay plate (Corning), and an equal volume (40 μL) of a colorimetric reagent was added (CytoTox 96; Promega) for 30 min at room temperature, with protection from light. The reaction mixture was quenched with an equal volume (40 μL) of 1 M acetic acid, and the absorbance at 490 nm was recorded using a SpectraMax M5 plate reader. The percent cell lysis was determined as follows: % lysis = 100% × [(*A*_490,sample_ − *A*_490,untreated cells_)/(*A*_490,lysis control_ − *A*_490,untreated cells_)].

### Bacterial strains and growth.

B. pertussis strain TohamaI and an isogenic derivative with a glycine-serine dipeptide insertion to inactivate ACT’s enzymatic activity, TohamaI/mutAC, were kind gifts from Peter Sebo (10). Cells were streaked from freezer stocks onto Bordet-Gengou agar plates (Becton, Dickinson) supplemented with 15% defibrinated sheep’s blood (Hemostat) and 0.7% glycerol and grown for 3 days at 37°C. Hemolytic bacterial colonies from one full plate were inoculated to an OD_600_ of ~0.05 in complete Stainer-Scholte medium with heptakis and grown for ~18 h at 37°C at 225 rpm. Challenge inocula were prepared as previously described (38). Briefly, liquid cultures grown overnight were concentrated ~5-fold and resuspended in complete SSM with 100% sterile glycerol to a final concentration of 15% (vol/vol) before freezing aliquots at −80°C. To determine the inoculum concentration, one aliquot was thawed, serially diluted in PBS, and plated dropwise onto BG plates in triplicate to determine the initial CFU per milliliter, which was typically ~2 × 10^10^ CFU/mL.

### Murine challenge experiments.

All animal procedures were performed in a facility accredited by the Association for Assessment and Accreditation of Laboratory Animal Care International according to protocols approved by UT Austin (protocol numbers 2015-00078 and 2018-00092) Animal Care and Use Committees and the principles outlined in the *Guide for the Care and Use of Laboratory Animals* (69). Prior to infection, 28- to 32-day-old BALB/c mice were weighed, anesthetized with 60 mg/kg of body weight of ketamine plus 8 mg/kg of xylazine, and administered 25 μL of saline or bacteria to each naris. In some cases, 5 μL antibody was first administered to each naris, followed by the administration of 20 μL bacteria to each naris. In other cases, mice were administered 200 μL sterile-filtered antibodies intraperitoneally 2 h before infection. Mice were placed on a heating pad, monitored until they regained consciousness, and returned to their cages.

Mouse welfare checks were performed four to five times daily for 3 days and then every 12 h for the remainder of the experiment, with mouse weights being measured daily. Mice were euthanized if they met all of the following criteria for two consecutive well checks: (i) >25% weight loss for female or >30% weight loss for male mice, (ii) a lack of grooming, (iii) a hunched posture with squinty eyes, and (iv) nonresponsiveness to stimuli. Mice were sacrificed when they met euthanasia criteria or at the termination of the experiment by CO_2_ asphyxiation and cervical dislocation. Lungs were removed and homogenized with Dounce tissue grinders (Kimble-Chase) in ~1 mL PBS to enumerate B. pertussis colonization levels. Samples were serially diluted in PBS and plated in 7-μL drops onto Bordet-Gengou agar plates for growth at 37°C for 3 days to quantify bacterial CFU.

### Pharmacokinetic experiments.

Antibody lung bioavailabilities were compared following different administration methods. First, one group of 5-week-old CD1 mice was intraperitoneally (i.p.) administered 100 μL of a 460-μg/mL solution of M2B10 in PBS (*n* = 3). A second group was sedated as described above, and 10 μL of a 2.3-mg/mL M2B10 solution was administered to each naris (*n* = 3). Seventeen hours after antibody administration, mice were sacrificed by cardiac puncture and cervical dislocation. Serum samples from cardiac puncture were collected, and the lungs were harvested. The lungs were homogenized with Dounce tissue grinders (Kimble-Chase) with Halt protease inhibitors (1:100 dilution; Pierce), and the supernatant was collected.

Antibody delivery by nasal drip was validated by comparison to aerosols delivered by a nebulizer. A mesh nebulizer (Aeroneb Solo; Aerogen) was used to generate aerosols. Samples of the input solution and flowthrough were collected, and ELISAs and SDS-PAGE were performed to determine the activity of the antibody after nebulization.

For mouse aerosol delivery studies, 4-week-old CD1 mice (Charles River) were acclimated to thve nose-only chamber. On days 2, 4, and 7, mice were placed into the chamber for a 10-min exposure to ambient air delivered at 1.5 L/min. On day 2, after mouse acclimation, albuterol and an antibody solution were run to determine the dose received by including filters at the nose-only inserts. For nasal drip, antibodies were applied in 10-μL drops to each naris, after anesthetizing the mice with a ketamine-xylazine anesthetic cocktail. Seventeen hours following antibody administration, all mice were sacrificed, and lung samples were collected as described above.
