# Supplementary figures and images for: Blockade of the Adenylate Cyclase Toxin Synergizes with Opsonizing Antibodies to Protect Mice against Bordetella pertussis
Source: mBio. 2022 Aug 3;13(4):e01527-22. doi: 10.1128/mbio.01527-22 (PMC9426472; doi:10.1128/mbio.01527-22)

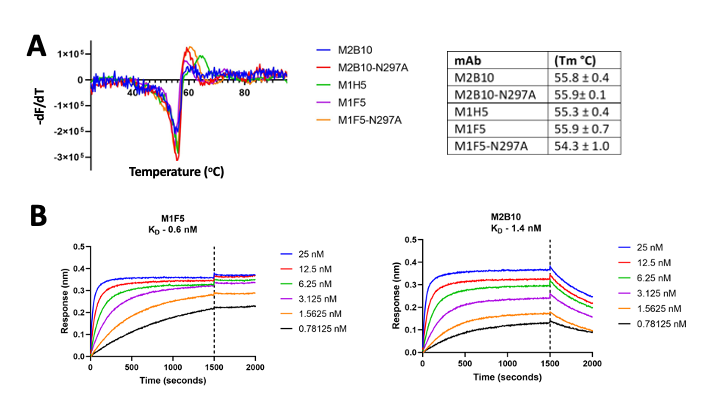

Supplement: FIG S1 [file mbio.01527-22-s0001.tif]

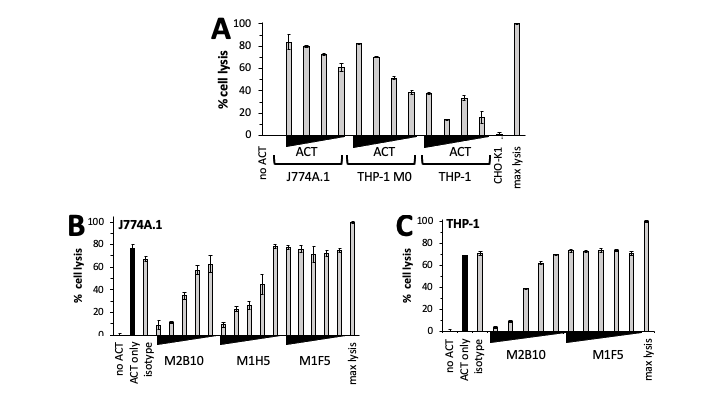

Supplement: FIG S2 [file mbio.01527-22-s0002.tif]

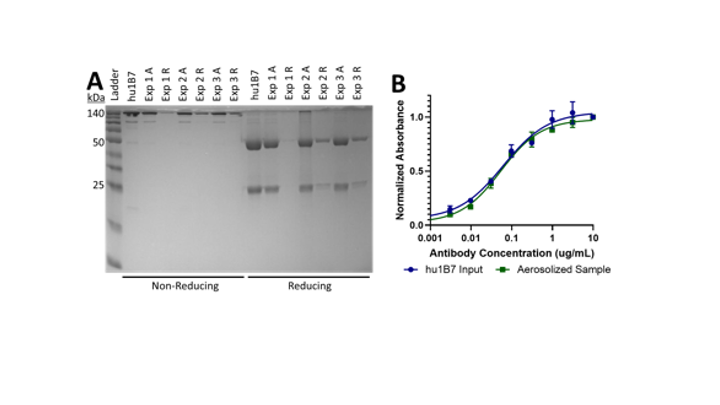

Supplement: FIG S3 [file mbio.01527-22-s0003.tif]

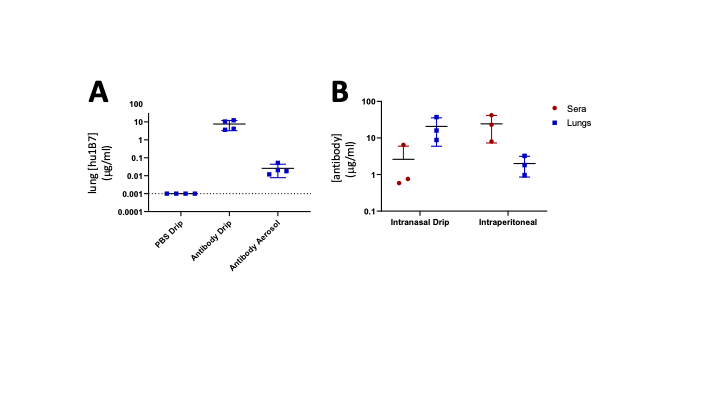

Supplement: FIG S4 [file mbio.01527-22-s0004.tif]

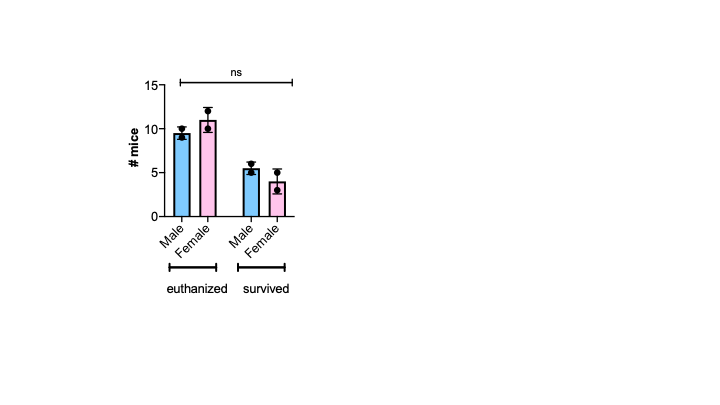

Supplement: FIG S5 [file mbio.01527-22-s0005.tif]
